# NusG-Dependent RNA Polymerase Pausing and Tylosin-Dependent Ribosome Stalling Are Required for Tylosin Resistance by Inducing 23S rRNA Methylation in Bacillus subtilis

**DOI:** 10.1128/mBio.02665-19

**Published:** 2019-11-12

**Authors:** Helen Yakhnin, Alexander V. Yakhnin, Brandon L. Mouery, Zachary F. Mandell, Catherine Karbasiafshar, Mikhail Kashlev, Paul Babitzke

**Affiliations:** a Department of Biochemistry and Molecular Biology, Pennsylvania State University, University Park, Pennsylvania, USA; b Center for RNA Molecular Biology, Pennsylvania State University, University Park, Pennsylvania, USA; c RNA Biology Laboratory, Center for Cancer Research, National Cancer Institute, Frederick, Maryland, USA; University of Minnesota Medical School

**Keywords:** NusG-dependent RNA polymerase pausing, transcription attenuation, antibiotic resistance, rRNA methylation

## Abstract

Macrolide antibiotics bind to 23S rRNA within the peptide exit tunnel of the ribosome, causing the translating ribosome to stall when an appropriately positioned macrolide arrest motif is encountered in the nascent polypeptide. Tylosin is a macrolide antibiotic produced by Streptomyces fradiae. Resistance to tylosin in S. fradiae is conferred by methylation of 23S rRNA by TlrD and RlmA^II^. Here, we demonstrate that *yxjB* encodes RlmA^II^ in Bacillus subtilis and that YxjB-specific methylation of 23S rRNA in the peptide exit tunnel confers tylosin resistance. Growth in the presence of subinhibitory concentrations of tylosin results in increased rRNA methylation and increased resistance. In the absence of tylosin, *yxjB* expression is repressed by transcription attenuation and translation attenuation mechanisms. Tylosin-dependent induction of *yxjB* expression relieves these two repression mechanisms. Induction requires tylosin-dependent ribosome stalling at an RYR arrest motif at the C terminus of a leader peptide encoded upstream of *yxjB*. Furthermore, NusG-dependent RNA polymerase pausing between the leader peptide and *yxjB* coding sequences is essential for tylosin-dependent induction. Pausing synchronizes the position of RNA polymerase with ribosome position such that the stalled ribosome prevents transcription termination and formation of an RNA structure that sequesters the *yxjB* ribosome binding site. On the basis of our results, we are renaming *yxjB* as *tlrB*.

## Abstract (precis)

Antibiotic resistance is a growing health concern. Resistance mechanisms have evolved that provide bacteria with a growth advantage in their natural habitat such as the soil. We determined that B. subtilis, a Gram-positive soil organism, has a mechanism of resistance to tylosin, a macrolide antibiotic commonly used in the meat industry. Tylosin induces expression of *yxjB*, which encodes an enzyme that methylates 23S rRNA. YxjB-dependent methylation of 23S rRNA confers tylosin resistance. NusG-dependent RNA polymerase pausing and tylosin-dependent ribosome stalling induce *yxjB* expression, and hence tylosin resistance, by preventing transcription termination upstream of the *yxjB* coding sequence and by preventing repression of *yxjB* translation.

## INTRODUCTION

Posttranscription initiation control mechanisms that regulate transcription termination and translation initiation represent common strategies that bacteria utilize to regulate gene expression in response to a variety of external stimuli (1–3). Regulated transcription termination in the leader region of operons dictates the extent to which RNA polymerase (RNAP) transcribes into the downstream genes. These transcription attenuation mechanisms typically involve overlapping antiterminator and terminator structures that can form in the nascent transcript (1). RNAP pausing can participate in transcription attenuation by providing sufficient time for RNA structure formation and/or regulatory factor binding (4–6). Regulation of translation initiation is another strategy used by bacteria to control gene expression (2, 7). RNA structures that sequester the Shine-Dalgarno (SD) sequence prevent ribosome binding, leading to translational repression.

During translation, polypeptides travel through the peptide exit tunnel (PET) within the large ribosomal subunit (7–9). Macrolide antibiotics bind exclusively to 23S rRNA within the PET (10). Once bound, these antibiotics can cause stalling of the translating ribosome when an appropriately positioned macrolide arrest motif is encountered in the nascent peptide. The known arrest motifs are typically three amino acids in length, with the downstream residue corresponding to the ribosome A site (11). Only a subset of any of the arrest motifs leads to translation arrest (11), suggesting that additional amino acids within the nascent polypeptide contribute to ribosome stalling (11, 12).

Resistance to a macrolide antibiotic can occur via methylation of its 23S rRNA target in the PET. For example, Erm methylates the N6 position of A2058, leading to resistance to several macrolides (13, 14). Gram-negative bacteria contain RlmA^I^, which methylates the N1 position of G745, whereas Gram-positive organisms contain RlmA^II^, which methylates the N1 position of G748. Tylosin is a macrolide antibiotic produced by Streptomyces fradiae. Synergistic high-level tylosin resistance in S. fradiae is conferred by methylation of A2058 by TlrD and by methylation of G748 by RlmA^II^ (15). Methylation of G748 is specific to tylosin resistance because the mycinose sugar moiety contacts this residue (14, 15).

We determined that *yxjB* encodes RlmA^II^ in B. subtilis, that YxjB-specific methylation of G748 confers tylosin resistance, and that tylosin induces *yxjB* expression. The induction mechanism requires NusG-dependent RNAP pausing in the *yxjB* leader as well as tylosin-dependent ribosome stalling during translation of a leader peptide, while translation of the leader peptide is required to reduce transcription termination in the *yxjB* leader region and to overcome a translation attenuation mechanism that represses YxjB synthesis.

## RESULTS

### *yxjB* encodes RlmA^II^ in B. subtilis.

**FIG 1:**
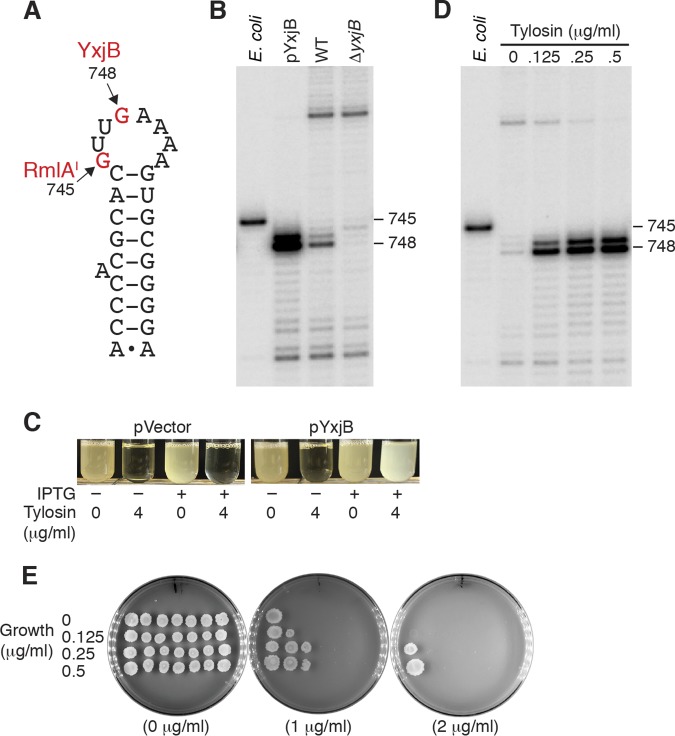
YxjB-mediated methylation of G748 in 23S rRNA confers resistance to tylosin. (A) Helix 35 of 23S rRNA. Positions of methylation by E. coli RmlA^I^ and B. subtilis YxjB are shown (E. coli numbering). (B) Primer extension inhibition was used to detect methylated residues in helix 35 of 23S rRNA in WT and Δ*yxjB* strains, as well as a strain in which *yxjB* was overexpressed from an IPTG-inducible promoter (pYxjB). Methylation of 23S rRNA from E. coli is shown as a control. (C) Overexpression of plasmid-borne *yxjB* from an IPTG-inducible promoter confers tylosin resistance. pVector was used as a control. (D) Primer extension inhibition was used to detect the position of methylated residues in 23S rRNA when cultures were grown in the presence of the indicated tylosin concentration. Methylation of 23S rRNA from E. coli was used as a control. (E) Growth in the presence of subinhibitory tylosin concentrations leads to increased tylosin resistance. Cultures were grown with the tylosin concentrations indicated at the left, and then serial dilutions were spotted onto plates containing the tylosin concentration shown below each plate. Experiments were performed at least twice with comparable results.

RlmA^I^ from Escherichia coli methylates G745 of 23S rRNA, while RlmA^II^ from Gram-positive bacteria methylates G748 within the same helix (14, 16). B. subtilis YxjB is 30% identical to RlmA^II^ from S. fradiae (16). To determine whether YxjB methylates G748 in B. subtilis, primer extension inhibition experiments were performed on RNA isolated from wild-type (WT), Δ*yxjB*, and *yxjB* overexpression strains. In this assay, reverse transcriptase (RT) terminates at the nucleotide preceding a residue containing a methyl group on the Watson-Crick (WC) face of the nucleobase. Similar experiments were carried out on RNA isolated from E. coli as a control. RT stops were observed at positions 749 and 746 on B. subtilis and E. coli 23S rRNA, respectively, indicating that 23S rRNA was methylated at position G748 in B. subtilis and position G745 in E. coli (Fig. 1A and B). Deletion of B. subtilis *yxjB* eliminated methylation at G748, while overexpression of *yxjB* increased methylation (Fig. 1B). On the basis of previous studies of other Gram-positive organisms, we conclude that *yxjB* encodes RlmA^II^ in B. subtilis.

In addition to G748, an RT stop corresponding to U747 was observed for B. subtilis (Fig. 1B). Deletion and overexpression of *yxjB* eliminated and increased the RT stops at the two positions, respectively. While we infer that the RT stops were caused by modification on the WC face of both nucleobases, we did not investigate the molecular basis for the RT stop at U747.

### YxjB-mediated methylation of G748 in 23S rRNA confers resistance to tylosin.

S. fradiae produces the macrolide antibiotic tylosin. Methylation of G748 by RlmA^II^ in S. fradiae confers low-level resistance to tylosin, whereas high-level resistance also requires TlrD-mediated methylation of A2058 in 23S rRNA (15). Both of these residues line the PET and, when methylated, inhibit tylosin interaction (14–16). Although B. subtilis does not produce tylosin, since both S. fradiae and B. subtilis are soil organisms, it was conceivable that B. subtilis had evolved resistance to tylosin produced by S. fradiae. We found that WT B. subtilis grew well with tylosin concentrations at or below 0.5 μg/ml and stopped growing above 1 μg/ml. To determine whether overexpression of *yxjB* would lead to increased resistance, strains containing the *yxjB* overexpression plasmid or an empty vector were grown with or without tylosin. While both strains grew in the absence of tylosin, induction of *yxjB* expression was required for growth in the presence of 4 μg/ml tylosin (Fig. 1C).

As *yxjB* overexpression resulted in increased methylation of G748 in 23S rRNA and resistance to tylosin, we reasoned that growth in the presence of subinhibitory tylosin concentrations could lead to increased methylation of this residue. Hence, primer extension was performed on 23S rRNA extracted from WT cells grown in the absence and presence of increasing tylosin concentrations. Methylation of G748 was greatly increased in the presence of the lowest concentration of tylosin tested, with methylation gradually increasing as the tylosin concentration was increased further (Fig. 1D).

We next tested whether growth in the presence of subinhibitory tylosin concentrations increased resistance to the antibiotic. The WT strain was grown in the absence or presence of 0.125, 0.25, or 0.5 μg/ml tylosin. Aliquots of 10-fold serial dilutions were then spotted onto plates containing 0, 1, or 2 μg/ml tylosin. The results of this analysis indicated that growth in the presence of subinhibitory concentrations of tylosin leads to increased resistance to this antibiotic (Fig. 1E). We conclude that growth in the presence of tylosin increases YxjB activity, leading to increased methylation of G748 in 23S rRNA and thereby conferring resistance to this antibiotic.

### A NusA-dependent terminator and an SD-sequestering hairpin are present in the *yxjB* leader.

**FIG 2:**
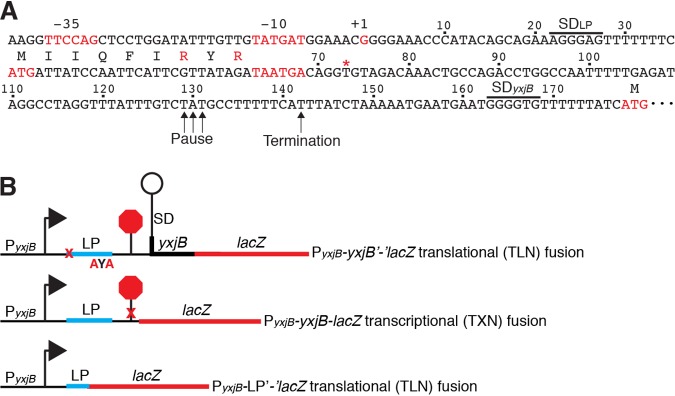
*yxjB* promoter and leader region and *yxjB-lacZ* fusions. (A) *yxjB* promoter and leader region. The −35 and −10 promoter elements, transcription start site (+1), translation initiation and stop codons of the leader peptide, and *yxjB* translation initiation codon are indicated in red. The leader peptide coding sequence and the leader peptide and *yxjB* SD sequences are shown. Termination and NusG-dependent pause sites are marked. The tylosin-dependent ribosome toeprint is marked with a red asterisk (*). Numbering is with respect to the start of *yxjB* transcription. (B) Schematic representation of the P*_yxjB_-yxjB-lacZ* transcriptional (TXN) fusion and of the P*_yxjB_-yxjB*′-′*lacZ* and P*_yxjB_-*LP′-′*lacZ* translational (TLN) fusions. The transcription start site is indicated with an arrow, and the leader peptide (LP) is indicated in cyan, the N-terminal *yxjB* coding sequence in black, and *lacZ* in red. The terminator (stop sign) and SD-sequestering hairpin are shown. Mutations in the terminator (X) or the leader peptide (X, AYA) are indicated in red.

**FIG 3:**
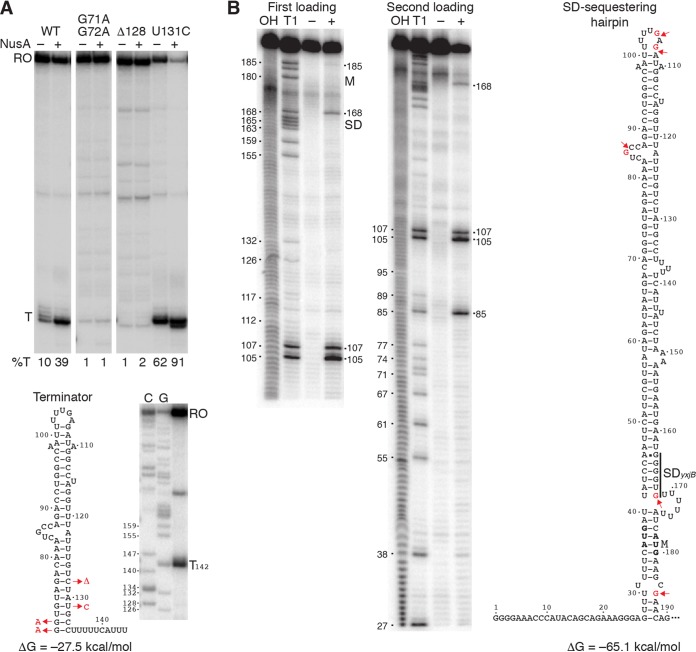
NusA-dependent terminator and SD-sequestering hairpin in the *yxjB* leader. (A) *In vitro* transcription termination assays using wild-type (WT) or mutant templates were performed in the absence (–) or presence (+) of NusA. Terminated (T) and runoff (RO) transcripts are marked. Percent termination is shown below each lane. The structure of the terminator is shown below with point mutations indicated in red. A termination assay adjacent to a sequencing ladder generated with 3′ dC or 3′ dG is also shown. Numbering is with respect to the start of *yxjB* transcription. (B) Structure mapping of the SD-sequestering hairpin. *yxjB* RNA was subjected to limited RNase T1 digestion (+). –, control without RNase T1. Partial alkaline hydrolysis (OH) and RNase T1 digestion (T1) ladders are shown. Residues that were cleaved by RNase T1 are indicated on the right. The numbering on the left corresponds to G residues in *yxjB*. The positions of the *yxjB* SD sequence and start codon (M) are marked. The structure of the *yxjB* SD-sequestering hairpin is on the right with cleaved G residues marked in red. Experiments were performed at least twice with comparable results.

Since growth in tylosin resulted in increased methylation of 23S rRNA and resistance to this antibiotic, we set out to determine the molecular mechanism(s) responsible for this phenomenon. Primer extension analysis led to the identification of a single *yxjB* transcription start site 177 nucleotides (nt) upstream of the *yxjB* translation initiation codon (see Fig. S1 in the supplemental material), which allowed us to identify a σ^A^-dependent promoter upstream (Fig. 2A). The presence of the 177-nt-long leader suggested that *yxjB* expression could be controlled posttranscriptionally. RNA structure predictions (17) identified a potential intrinsic transcription terminator in the *yxjB* leader region, suggesting that *yxjB* expression could be controlled by transcription attenuation (Fig. 3A). However, an overlapping antiterminator was not identified. Additional computer modeling led to the prediction that transcripts that failed to terminate would form a structure that would sequester the *yxjB* SD sequence and initially translated region, suggesting that *yxjB* could also be under the control of a translational regulatory mechanism (Fig. 3B).

10.1128/mBio.02665-19.2 FIG S1 Primer extension mapping of the *yxjB* transcription start site. Primer extension analysis was performed on total cellular RNA extracted from a wild-type strain of B. subtilis. The primer extension (PE) product is indicated by a red asterisk (*). Sequence surrounding the transcription start site (G) is shown on the left. The arrow indicates the direction of transcription. Sequencing lanes (A, C, G, and T) are shown. Download FIG S1, TIF file, 0.4 MB. Copyright © 2019 Yakhnin et al. 2019 Yakhnin et al. This content is distributed under the terms of the Creative Commons Attribution 4.0 International license.

Results from *in vitro* transcription experiments demonstrated that termination occurred over a 4-nt window, with the strongest termination occurring at position +142. Although the basal termination efficiency was low, the addition of NusA, a general transcription elongation factor known to stimulate intrinsic termination *in vitro* and *in vivo* (18), resulted in a 4-fold increase in termination (Fig. 3A). We also tested the effect of terminator mutations predicted to strengthen or weaken the terminator. The G71A:G72A mutant resulted in a shortened terminator hairpin followed by two C residues. These substitutions virtually eliminated termination (Fig. 3A), consistent with previous observations that U residues immediately downstream from the hairpin are crucial (18, 19). Similarly, deleting C128, which eliminated a G-C base pair and introduced a single nucleotide bulge in the hairpin, resulted in a severe termination defect. In contrast, the U131C mutation strengthened the hairpin by replacing a G-U with a G-C base pair, resulting in more-efficient termination (Fig. 3A). Importantly, this NusA-dependent terminator could be involved in a transcription attenuation mechanism.

*In vitro* RNA structure mapping experiments were also conducted to determine whether the *yxjB* SD-sequestering hairpin formed as predicted. A transcript extending from +1 to +190 was subjected to limited RNase T1 digestion, which cleaves RNA following single-stranded G residues. We observed strong cleavage at positions G85, G105, G107, and G168 and weak cleavage at G185 (Fig. 3B). These results are consistent with the structure predicted by Mfold, with four exceptions. An apparently noncanonical A46-G163 base pair forms (20), while the U30-G185, U41-G168, and U101-G107 base pairs do not form. Importantly, this structure would be capable of repressing *yxjB* translation (Fig. 3B).

### Tylosin is required for induction of *yxjB* expression.

P*_yxjB_*-*yxjB-lacZ* transcriptional and P*_yxjB_*-*yxjB*′-′*lacZ* translational fusions were constructed and integrated into the chromosomal *amyE* locus to examine *yxjB* expression. Both fusions would be subject to transcription attenuation, while the translational fusion would also be subject to any mechanism regulating *yxjB* translation (Fig. 2B). Expression of the transcriptional fusion was observed throughout growth, with the highest level of expression occurring during the transition to and within stationary phase (Table 1, row 1). Introduction of the G71A and G72A substitutions that eliminated termination *in vitro* resulted in 2-fold-higher expression (Table 1, rows 1 and 2). In contrast to the transcriptional fusion results, expression of the translational fusion was not observed (Table 1, row 4). The latter result was consistent with the possibility that translation was repressed by an SD-sequestering hairpin.

**TABLE 1:** Effects of termination, tylosin, leader peptide, and pausing on *yxjB* expression

Row	Fusion^*b*^	Strain	Tyl	Ery^*c*^	β-Galactosidase activity^*a*^			
					Mid-exp	Late-exp	Transition	Stationary
1	*yxjB-lacZ* TXN	WT	0	0	11 ± 2	15 ± 3	20 ± 3	21 ± 3
2	*yxjB-lacZ* TXN T-mut	WT	0	0	17 ± 2	33 ± 3	40 ± 5	40 ± 5
3	*yxjB-lacZ* TXN	WT	0.5	0	28 ± 4	31 ± 3	41 ± 4	41 ± 5
4	*yxjB*′-′*lacZ* TLN	WT	0	0	<1	<1	<1	1 ± 0.1
5	*yxjB*′*-′lacZ* TLN	WT	0.125	0	<1	2 ± 0.2	6 ± 0	10 ± 1
6	*yxjB*′-′*lacZ* TLN	WT	0.25	0	4 ± 1	5 ± 0.5	10 ± 2	13 ± 3
7	*yxjB*′-′*lacZ* TLN	WT	0.5	0	5 ± 1	8 ± 3	14 ± 3	17 ± 1
8	*yxjB*′-′*lacZ* TLN	WT	0	0.05	<1	<1	1 ± 0.1	1 ± 0.3
9	LP′-′*lacZ* TLN	WT	0	0	3 ± 0.1	6 ± 0.2	7 ± 0.2	9 ± 0.2
10	*yxjB*′-′*lacZ* TLN LP-mut	WT	0	0	<1	<1	<1	1 ± 0.1
11	*yxjB*′-′*lacZ* TLN LP-mut	WT	0.5	0	<1	<1	<1	<1
12	*yxjB*′-′*lacZ* TLN LP-AYA	WT	0	0	<1	<1	2 ± 0.4	2 ± 0.6
13	*yxjB*′-′*lacZ* TLN LP-AYA	WT	0.5	0	<1	<1	2 ± 0.4	2 ± 0.4
14	*yxjB*′-′*lacZ* TLN	Bacillus subtilis Δ*nusG*	0	0	<1	<1	1 ± 0.4	2 ± 0.2
15	*yxjB*′-′*lacZ* TLN	Bacillus subtilis Δ*nusG*	0.5	0	<1	<1	3 ± 0.2	3 ± 0.2
16	*yxjB*′-′*lacZ* TLN P-mut	WT	0	0	<1	<1	<1	<1
17	*yxjB*′-′*lacZ* TLN P-mut	WT	0.5	0	1 ± 0.1	3 ± 0.5	5 ± 0.5	6 ± 0.1

a β-Galactosidase activity was measured during the mid-exponential phase (Mid-exp), the late exponential phase (Late-exp), the transition between exponential and stationary phase (Transition), and the stationary phase (Stationary). Cells were grown in the absence or presence of the indicated concentration (in micrograms per milliliter) of tylosin (Tyl) or erythromycin (Ery). Each experiment was performed at least 3 times. Values are given in Miller units ± standard deviations.

b *yxjB-lacZ* TXN, P*_yxjB_*-*yxjB-lacZ* transcriptional fusion; *yxjB-lacZ* TXN T-mut, P*_yxjB_*-*yxjB-lacZ* transcriptional fusion with G71A and G72A terminator mutations; *yxjB*′-′*lacZ* TLN, P*_yxjB_*-*yxjB*′-′*lacZ* translational fusion; LP′-′*lacZ* TLN, P*_yxjB_*-LP′-′*lacZ* translational fusion; *yxjB*′-′*lacZ* TLN LP-mut, P*_yxjB_*-*yxjB*′-′*lacZ* translational fusion with the LP ATG start codon mutated to ACG; *yxjB*′-′*lacZ* TLN LP-AYA, P*_yxjB_*-*yxjB*′-′*lacZ* translational fusion with the LP RYR ribosome stalling motif mutated to AYA; *yxjB*′-′*lacZ* TLN P-mut, P*_yxjB_*-*yxjB*′-′*lacZ* translational fusion with the T131A pause site mutation.

c No measurable expression was observed when cells were grown in the presence of erythromycin at 0.0125, 0.025, or 0.05 μg/ml. Data are shown for the highest concentration only.

The lack of expression of the translational fusion led us to test whether there was an interrelationship between tylosin and *yxjB* expression. Thus, the P*_yxjB_*-*yxjB*′-′*lacZ* translational fusion strain was grown in the presence of subinhibitory concentrations of tylosin. Expression of this fusion was observed at the lowest concentration of tylosin tested, while expression gradually increased as the concentration of tylosin was increased (Table 1, rows 4 to 7). Remarkably, the gradual increase in expression correlated precisely with the gradual increase in 23S rRNA methylation observed under the same growth conditions (Fig. 1D). Expression of the transcriptional fusion also increased about 2-fold in the presence of tylosin (Table 1, rows 1 and 3). We conclude that tylosin induces *yxjB* expression and that tylosin is required for expression when the entire leader region is present, as is the case for the translational fusion.

To determine whether induction of *yxjB* expression was a general feature of macrolide antibiotics, we tested the effect of erythromycin. We found that cells grew well in the presence of 0.05 μg/ml erythromycin but grew poorly at a concentration of 0.1 μg/ml. Expression of the P*_yxjB_*-*yxjB*′-′*lacZ* translational fusion was not induced when cells were grown in the presence of subinhibitory concentrations of erythromycin (Table 1, rows 4 and 8), indicating that induction of *yxjB* expression is not a general feature of macrolide antibiotics and may be specific to tylosin.

### Tylosin-dependent induction of *yxjB* expression requires translation of a leader peptide.

The results described above indicate that tylosin-dependent induction of *yxjB* expression leads to increased methylation of 23S rRNA, thereby conferring resistance to tylosin. In addition to the terminator and SD-sequestering hairpin, we identified a 9-amino-acid open reading frame in the *yxjB* leader. This coding sequence was preceded by an SD sequence and followed by tandem termination codons (Fig. 2A). Thus, expression of a chromosomally integrated P*_yxjB_*-LP′-′*lacZ* translational fusion was examined to determine whether the leader peptide was expressed (Fig. 2B). We found that this fusion was expressed and that expression increased throughout growth (Table 1, row 9). We then introduced an ATG-to-ACG mutation in the start codon of the leader peptide in the context of the P*_yxjB_*-*yxjB*′-′*lacZ* translational fusion to determine whether expression of the leader peptide affected *yxjB* expression. Preventing translation of the leader peptide by this mutation eliminated tylosin-dependent induction of *yxjB* expression (Table 1; compare rows 4 and 7 with rows 10 and 11). We conclude that translation of the leader peptide is required for tylosin-dependent induction of *yxjB* expression.

### Tylosin-dependent ribosome stalling during leader peptide synthesis is required for *yxjB* expression.

Macrolide antibiotics bind within the PET of the ribosome (9, 10). In conjunction with specific sequence motifs within the nascent peptide, bound macrolides are capable of causing ribosome stalling. These stalling sites are enriched in prolines and charged residues and can be found throughout the coding region (11). One such stalling motif, R/K-X-R/K, was identified in a transcriptomic study investigating the effect of macrolides in Staphylococcus aureus (11). The last three residues of the *yxjB* leader peptide (RYR) match this motif (Fig. 2A). To determine whether this motif was involved in tylosin-dependent induction, nucleotide substitutions were introduced into the P*_yxjB_*-*yxjB*′-′*lacZ* translational fusion such that the RYR motif was changed to AYA. Other than generating an internal loop consisting of C54 to G61 and C147 to G155, these substitutions did not affect the predicted structure of the SD-sequestering hairpin (Fig. 3B). Importantly, this mutation virtually eliminated tylosin-dependent induction (Table 1; compare rows 4 and 7 with rows 12 and 13), suggesting that tylosin-dependent ribosome stalling is responsible for induction of *yxjB* expression.

**FIG 4:**
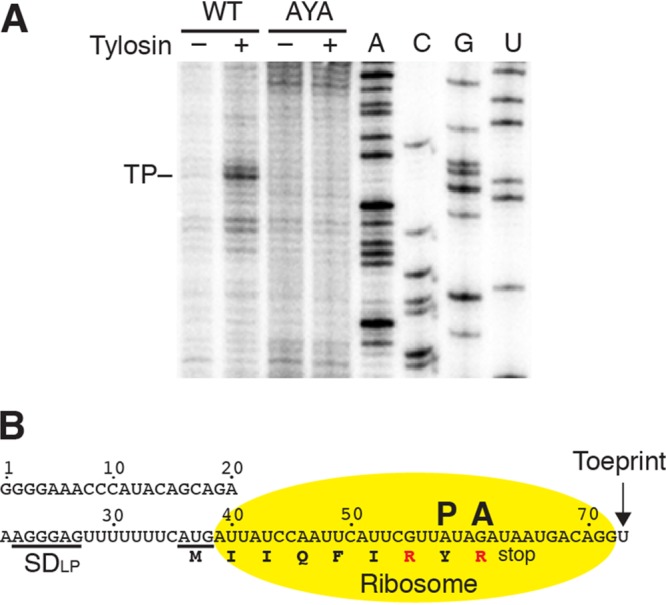
Tylosin-dependent ribosome stalling in the leader peptide. (A) Toeprint analysis of tylosin-induced ribosome stalling during translation of the leader peptide using WT and AYA mutant templates. The toeprint (TP) identified with the WT template in the presence of tylosin (+) is marked. Sequencing lanes (A, C, G, and U) are shown. The PURExpress kit containing T7 RNAP and E. coli ribosomes was used for this analysis. (B) *yxjB* leader region covered by the ribosome when tylosin induces stalling. The positions of the toeprint and the ribosome peptidyl (P) and aminoacyl (A) sites are shown. Additional details are as described in the Fig. 2A legend. Experiments were performed at least twice with comparable results.

We next conducted an *in vitro* toeprinting assay using a coupled transcription-translation assay to provide direct evidence for tylosin-dependent ribosome stalling during leader peptide synthesis. The position of stalled ribosomes was detected by primer extension inhibition. A tylosin-dependent toeprint was identified at position U74 in the WT *yxjB* leader but not in the AYA mutant (Fig. 4A). A toeprint was not observed when experiments were carried out with erythromycin (data not shown). Prior toeprinting studies performed with 30S ribosomal subunits identified toeprints 15 to 16 nt downstream from the A of AUG initiation codons (11, 21). This distance places the tyrosine and the second arginine of the RYR motif at the P and A sites of the stalled ribosome, respectively (Fig. 4B). We conclude that induction of *yxjB* expression requires tylosin-dependent ribosome stalling at the RYR motif of the leader peptide.

### NusG-dependent RNAP pausing is required for tylosin-dependent induction of *yxjB* expression.

The position of the stalled ribosome provides an explanation for how tylosin induces *yxjB* expression. A ribosome stalled at this position would prevent completion of the terminator hairpin, resulting in transcriptional readthrough. Moreover, the stalled ribosome would prevent formation of the *yxjB* SD sequestering hairpin such that the *yxjB* SD sequence would be available for ribosome binding (Fig. 3B and 4B). This issue poses a dilemma because translation initiation of the leader peptide must occur before RNAP finishes transcribing the entire SD-sequestering hairpin, as this structure would also inhibit translation of the leader peptide. Thus, the position of actively transcribing RNAP and the timing of leader peptide translation would be critical for this regulatory mechanism. One way to circumvent this potential problem would be for RNAP to pause at an appropriate position during transcription of the *yxjB* leader region and thereby provide sufficient time for initiation of leader peptide translation.

**FIG 5:**
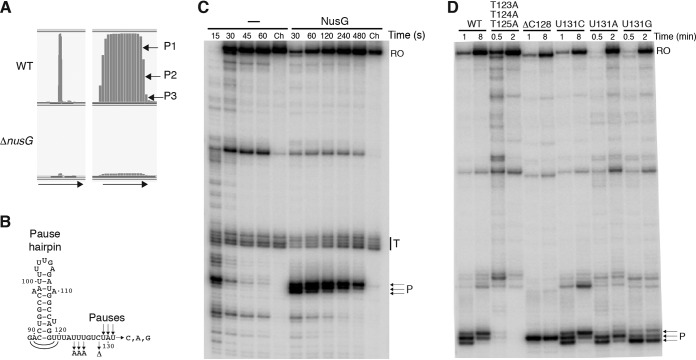
NusG-dependent pausing in the *yxjB* leader. (A) NusG-dependent RNAP pause sites identified by RNET-seq were observed only in the wild-type (WT) strain. Arrows indicate the direction of transcription. The right panel shows a zoomed image of the left panel. (B) Predicted *yxjB* pause hairpin structure for the first pause (U129). The pause hairpin likely extends one base pair for the second pause (A130) and two base pairs for the third pause (U131). Pause mutations are indicated with arrows. (C) *In vitro* transcription pausing assay performed in the absence (–) or presence of NusG. The time of the reaction is shown at the top of each lane. Ch, chase reactions. The positions of the same NusG-dependent pause sites (P) identified *in vivo* are indicated. Runoff (RO) and terminated (T) transcripts are marked. Experiments were performed at least twice with comparable results. (D) Effects of pausing mutants on NusG-dependent pausing *in vitro*. A single-round *in vitro* transcription pausing assay was performed in the presence of NusG using WT or the indicated mutant DNA templates.

Because of our interest in NusG-dependent pausing, we identified NusG-dependent pause sites throughout the B. subtilis transcriptome (A. Yakhnin, M. Kashkev, and P. Babitzke, unpublished data). This method, which combines native elongating transcript sequencing (NET-seq) with RNase footprinting of the transcripts and is called RNET-seq (22), identified three adjacent NusG-dependent pause sites in the *yxjB* leader that are preceded by an appropriately positioned pause hairpin (Fig. 2A) (see also Fig. 5A and B). Note that the pause hairpin forms the apex of the terminator and SD-sequestering hairpins (Fig. 3). Importantly, pausing at these positions (U129, A130, and U131) could provide sufficient time for initiation of leader peptide translation. Interestingly, previous Term-seq studies identified what was assumed to be a terminator in the *yxjB* leader, when in fact the identified 3′ ends actually correspond to the nascent 3′ end of the stable NusG-dependent pause site (18, 23).

*In vitro* transcription pausing assays were performed to obtain additional information on the NusG-dependent pause sites identified *in vivo*. Paused RNAP is visualized in this assay by an initial accumulation of a band corresponding to the paused transcription complex and then subsequent elongation to longer transcripts (24). We observed *in vitro* the same three pause sites as were identified by RNET-seq, and NusG greatly stimulated pausing at each position (Fig. 5C). The time course of the pausing assay indicated that RNAP paused first at position U129, then a second time at A130, and then a third time at U131.

Our prior studies on the *trp* leader NusG-dependent pause site demonstrated that NusG contacts a T-rich region within the nontemplate DNA strand of the paused transcription bubble (24–28). On the basis of our prior studies, we tested the effect of mutations that were predicted to interfere with NusG-dependent pausing in the *yxjB* leader (Fig. 5B and D). Simultaneously changing T123, T124, and T125 to A residues eliminated NusG-dependent pausing, while deletion of C128 resulted in a single pause at the position corresponding to U131. Changing U131 to C had no effect on pausing, whereas A and G substitutions virtually eliminated pausing at this position.

We compared the levels of expression of the P*_yxjB_*-*yxjB*′-′*lacZ* translational fusion in WT and Δ*nusG* strains and found that tylosin-dependent induction was nearly abolished in the Δ*nusG* strain (Table 1; compare rows 4 and 7 with rows 14 and 15). We also tested the effect of the T131A mutation on expression of the fusion. Consistent with our *in vitro* results (Fig. 5D), the T131A mutation reduced, but did not eliminate, tylosin-dependent induction (Table 1; compare rows 4 and 7 with rows 16 and 17). We conclude that NusG-dependent RNAP pausing contributes to tylosin-dependent induction of *yxjB* expression. We further infer that pausing provides sufficient time for translation of the leader peptide before the *yxjB* SD-sequestering hairpin has a chance to form.

## DISCUSSION

Macrolide antibiotics bind within the PET of the translating ribosome and contribute to ribosome stalling when an appropriate macrolide arrest motif is encountered, such as R/K-X-R/K. In the general translation attenuation mechanism, macrolide-dependent ribosome stalling during leader peptide synthesis disrupts a downstream SD-sequestering hairpin for the cognate resistance gene, thereby leading to specific antibiotic-induced expression. This general mechanism is thought to control expression of several macrolide resistance genes, including 23S rRNA methyltransferases, efflux proteins, and enzymes that inactivate the antibiotic (9). A similar translation attenuation mechanism controls expression of a gene conferring resistance to chloramphenicol (8, 29). A recent transcriptomics study in Listeria monocytogenes identified an antibiotic resistance mechanism that appears to combine macrolide-dependent ribosome stalling and transcription attenuation to control expression of a putative ribosome-splitting factor (30). Our results establish that *yxjB* encodes RlmA^II^, an enzyme that methylates G748 of 23S rRNA, and that modification of this residue confers resistance to tylosin (Fig. 1). Thus, we are renaming B. subtilis *yxjB* as *tlrB* to be consistent with established nomenclature (15).

**FIG 6:**
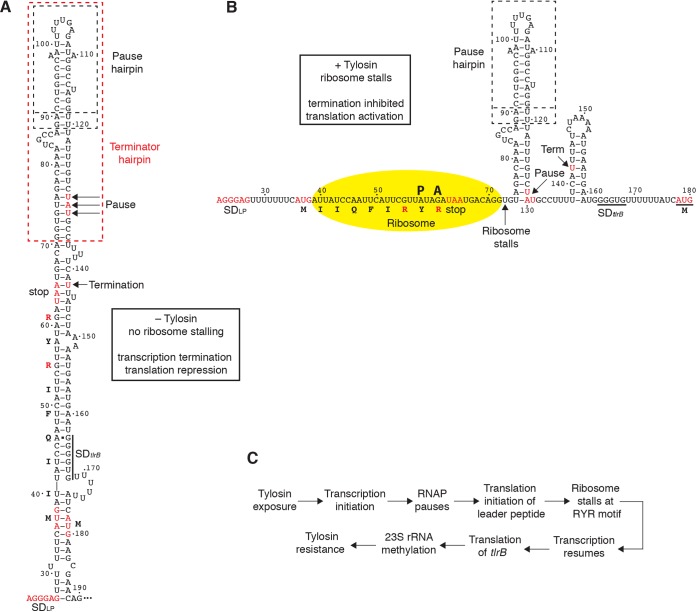
Model of tylosin-dependent induction of *tlrB* expression. (A) RNAP pauses at positions U129 to U131, providing time for translation initiation of the leader peptide. Translation of the leader peptide might disrupt the paused transcription elongation complex such that RNAP resumes transcription. In the absence of tylosin, the ribosome releases at the tandem stop codons. Once RNAP reaches the *tlrB* coding sequence, the entire *tlrB* SD-sequestering hairpin forms and represses *tlrB* translation. This hairpin also represses further rounds of leader peptide translation. (B) RNAP pausing provides time for translation initiation of the leader peptide. In the presence of tylosin, the ribosome stalls at the RYR motif such the ribosome remains bound to the nascent transcript. Once RNAP resumes transcription, the *tlrB* SD sequence is single stranded and translation is activated. RlmA^II^ then methylates G748 in 23S rRNA, leading to tylosin resistance. (C) Schematic representation of the series of events beginning with exposure to tylosin and culminating in tylosin resistance.

Regulation of B. subtilis *tlrB* expression combines tylosin-dependent ribosome stalling, transcription attenuation, and translation attenuation mechanisms. The mechanism regulating B. subtilis *tlrB* expression is far more complex than what has been described for other macrolide-induced ribosome stalling mechanisms. In addition to transcription attenuation and translation attenuation, NusG-dependent RNAP pausing is required for tylosin-dependent induction (Fig. 5) (Table 1). Perhaps RNAP pausing participates in other macrolide-dependent induction mechanisms. Our results are consistent with the following regulatory model (Fig. 6). In the absence of tylosin, NusG-dependent pausing provides sufficient time for translation initiation of the *tlrB* leader peptide. Once the ribosome approaches the stop codon, it is able to interfere with formation of the terminator hairpin provided that RNAP has escaped the pause state and resumed transcription. However, once translation of the leader peptide is completed, the released ribosome is no longer able to interfere with termination. Readthrough transcripts that fail to terminate would be subject to translation attenuation because the *tlrB* SD-sequestering hairpin would prevent ribosome binding. The combined effects of transcription termination and translation repression result in very low levels of RlmA^II^ synthesis. Although expression of the translational fusion was not detectable when cells were grown in the absence of tylosin (Table 1), some RlmA^II^ must be produced under this condition because G748 modification was detected when cells were grown without tylosin (Fig. 1B). *tlrB* expression is induced when cells are grown in the presence of subinhibitory concentrations of tylosin. In this case, RNAP pausing provides time for translation initiation of the leader peptide. Once the translating ribosome reaches the RYR motif in the leader peptide, the combination of tylosin bound to 23S rRNA in the PET and the RYR motif in the peptidyltransferase center causes the ribosome to stall prior to incorporation of the final arginine residue positioned at the ribosome A site. The positive charge of the two arginine residues and the length of the side chains interfere with peptide bond formation (31). The stalled ribosome would prevent transcription termination because nucleotides that are required for forming the base of the terminator hairpin would be bound by the stalled ribosome. Furthermore, the stalled ribosome would prevent formation of the SD-sequestering hairpin and thereby alleviate translational repression. The induced RlmA^II^ levels would lead to increased methylation of G748 in 23S rRNA and to tylosin resistance. Tylosin-dependent induction is self-limiting, since ribosomes modified at G748 would not stall during translation of the leader peptide, resulting in tight control of the level of 23S rRNA modification. Since there is no known enzyme capable of removing the methyl on G748, the level of methylation of cells no longer exposed to tylosin would slowly diminish as the organism continued to grow. This regulatory system ensures that methylation of 23S rRNA occurs only when B. subtilis comes in contact with tylosin. Presumably, the tight control of *tlrB* expression minimizes a fitness cost associated with methylation of G748 when tylosin is not present.

The complex regulatory mechanism for tylosin resistance in B. subtilis appears to be conserved in other *Bacillus* species (see Fig. S2 in the supplemental material). In each case, the intrinsic terminator, the *tlrB* SD-sequestering hairpin, the general position and length of the leader peptide, and the RXR motif are conserved. Potential NusG-dependent pause sites were also identified. The most significant difference in the three RNA structures is that the leader peptide SD sequence in B. pumilus is predicted to be sequestered in a weak hairpin.

10.1128/mBio.02665-19.3 FIG S2 Conservation of the tylosin-dependent induction mechanism. RNA structures predicted to form in the *tlrB* leader region of B. licheniformis and B. pumilus were compared to that of B. subtilis. The positions of the presumed leader peptide SD sequence (SD_LP_), leader peptide start (M), and stop codons, *tlrB* SD sequence (SD*_tlrB_*), and *tlrB* start codons (M) are shown for each organism. Conserved ribosome stalling sites in the three leaders, as well as RNAP pause and termination sites, are also indicated. Note that the ribosome stall site is positioned directly across from the RNAP pause sites in each structure. Numbering is relative to the presumed start of transcription. Download FIG S2, TIF file, 0.9 MB. Copyright © 2019 Yakhnin et al. 2019 Yakhnin et al. This content is distributed under the terms of the Creative Commons Attribution 4.0 International license.

Only a fraction of arrest motifs leads to macrolide-dependent ribosome stalling, indicating that additional nascent peptide residues traversing the PET participate in the stalling mechanism (11, 12). This context-dependent stalling is critical for regulating expression of resistance genes, which are often specific for a particular antibiotic (9). For example, a previous study demonstrated that erythromycin is capable of causing ribosome stalling at only a subset of R/K-X-R/K motifs (11). The inability of erythromycin to cause stalling at the RYR motif in the *tlrB* leader peptide provides further evidence that additional sequence information on the nascent peptide is required for the specificity of macrolide-induced ribosome stalling. Since the B. subtilis *tlrB* leader peptide is only 9 amino acids in length (Fig. 2A), when the ribosome is stalled the entire nascent peptide resides within the PET. The leader peptide from B. licheniformis is identical in sequence to that from B. subtilis; however, the leader peptide in B. pumilus differs in two positions. Hence, we generated a maximum likelihood phylogeny from a multiple-sequence alignment of 180 leader peptides predicted to be located directly upstream of *tlrB* homologs in Gram-positive organisms and to contain the R/K-X-R/K stalling motif at the C terminus (Fig. S3 and S4). The leader peptides are identical in several species, suggesting that the tylosin-dependent stalling mechanism is conserved in these organisms. It is likely that at least one of the first 6 amino acids in the leader peptide sequence (MIIQFIRYR) provides specificity for tylosin-dependent stalling. Perhaps as the leader peptide sequence diverges, the specificity of induction changes to include additional or alternative macrolides. B. subtilis, S. fradiae, and several other organisms in our phylogenetic analysis are soil microbes. The ability of other species to protect themselves from the harmful effects of tylosin produced by S. fradiae would provide a distinct growth advantage relative to other organisms that are incapable of inducing resistance to this antibiotic.

10.1128/mBio.02665-19.4 FIG S3 Phylogenetic conservation of the *tlrB* leader peptide. Maximum likelihood phylogeny data derived from a multiple-sequence alignment of leader peptides that contain the R/K-X-R/K stalling motif at the C terminus identified upstream of *yxjB* (*tlrB*) homologues in 180 Gram-positive bacterial species are presented. Leader peptide sequences of the organisms depicted with an asterisk are shown in Fig. S4. Download FIG S3, TIF file, 2.4 MB. Copyright © 2019 Yakhnin et al. 2019 Yakhnin et al. This content is distributed under the terms of the Creative Commons Attribution 4.0 International license.

10.1128/mBio.02665-19.5 FIG S4 Amino acid conservation of the *tlrB* leader peptide. Results of multiple-sequence alignment of leader peptides from 36 organisms shown in Fig. S3 are presented. Amino acids that are identical to those from B. subtilis are in red font, while those in which R residues in the RYR motif were replaced with K residues appear in cyan. The length of each leader peptide is indicated on the right. Download FIG S4, TIF file, 1.0 MB. Copyright © 2019 Yakhnin et al. 2019 Yakhnin et al. This content is distributed under the terms of the Creative Commons Attribution 4.0 International license.

## MATERIALS AND METHODS

### Bacterial strains and plasmids.

The B. subtilis strains used in this study are listed in Table 2, the plasmids used are listed in Table S1 in the supplemental material, and the oligonucleotides used are listed in Table S2. Strain and plasmid constructions are described in Text S1 in the supplemental material.

**TABLE 2:** B. subtilis strains used in this study

Strain	Genotype^*a*^	Source or reference
BKE39010	*yxjB*::*erm trpC2* Em^r^	BGSC^*b*^
PLBS338	Prototroph	21
PLBS538	*nusG*::*kan* Km^r^	23
PLBS800	*amyE*::P*_yxjB_-yxjB*′-′*lacZ* (−396 to +222) Cm^r^	This study
PLBS852	*yxjB*::*erm* Em^r^	This study
PLBS867	PLBS338/pYxjB Tc^r^	This study
PLBS868	PLBS338/pVector Tc^r^	This study
PLBS877	*amyE*::P*_yxjB_*-*yxjB-lacZ* (−396 to +153) Cm^r^	This study
PLBS952	*amyE*::P*_yxjB_*-LP'-'*lacZ* (−396 to +44) Cm^r^	This study
PLBS954	*amyE*::P*_yxjB_-yxjB*′-′*lacZ* (−396 to +222) *nusG*::*kan* Cm^r^ Km^r^	This study
PLBS957	*amyE*::P*_yxjB_-yxjB*′-′*lacZ* (−396 to +222, T37C) Cm^r^	This study
PLBS959	*amyE*::P*_yxjB_-yxjB*′-′*lacZ* (−396 to +222, T131A) Cm^r^	This study
PLBS960	*amyE*::P*_yxjB_*-*yxjB-lacZ* (−396 to +153, G71A:G72A) Cm^r^	This study
PLBS964	*amyE*::P*_yxjB_-yxjB*′-′*lacZ* (−396 to +222, C54G:G55C:T56C:A60G:G61C) Cm^r^	This study

a Numbers in parentheses indicate the cloned *yxjB* region relative to the start of transcription, as well as *yxjB* leader mutations. Em, erythromycin; Km, kanamycin; Cm, chloramphenicol; Tc, tetracycline.

b BGSC, *Bacillus* Genetic Stock Center.

10.1128/mBio.02665-19.1 TEXT S1 Supplemental materials and methods and corresponding references. Download Text S1, DOCX file, 0.02 MB. Copyright © 2019 Yakhnin et al. 2019 Yakhnin et al. This content is distributed under the terms of the Creative Commons Attribution 4.0 International license.

10.1128/mBio.02665-19.6 TABLE S1 Plasmids used in this study. Download Table S1, DOCX file, 0.02 MB. Copyright © 2019 Yakhnin et al. 2019 Yakhnin et al. This content is distributed under the terms of the Creative Commons Attribution 4.0 International license.

10.1128/mBio.02665-19.7 TABLE S2 Oligonucleotides used in this study. Download Table S2, DOCX file, 0.02 MB. Copyright © 2019 Yakhnin et al. 2019 Yakhnin et al. This content is distributed under the terms of the Creative Commons Attribution 4.0 International license.

### Primer extension assays.

For determination of the *yxjB* transcription start site, RNA was isolated from a late-exponential-phase culture of B. subtilis PLBS338 grown in LB. RNA was hybridized to a ^32^P-end-labeled oligonucleotide complementary to the *yxjB* leader. Primer extension reaction mixtures were incubated for 15 min at 42°C using SuperScript III reverse transcriptase (Thermo Fisher Scientific). Samples were denatured by heating for 5 min at 90°C and then fractionated through 6% polyacrylamide sequencing gels. Radiolabeled bands were imaged on a Typhoon 8600 Phosphorimager (GE Healthcare Life Sciences).

For identification of 23S rRNA methylation sites, RNA was isolated from B. subtilis strains PLBS338, PLBS852, and PLBS867, and E. coli *strain* MG1655 was grown in LB. RNA and hybridized to a ^32^P-end-labeled oligonucleotide complementary to nt 798 to 818 of E. coli 23S rRNA and to nt 845 to 865 of B. subtilis 23S rRNA. RT reaction procedures were identical to those described above except that primer extension was performed for 30 min. Details of the procedures are described in Text S1.

### Transcription termination and pausing assays.

Analysis of RNAP pausing was performed as described previously (24, 32) with modifications. Briefly, DNA templates contained WT or mutant *yxjB* leader sequences driven by a σ^A^ promoter as well as a 29-nt C-less cassette. Halted elongation complexes were formed by the exclusion of CTP. Elongation was resumed by the addition of all four nucleoside triphosphates (NTPs) (final concentration, 150 μM) and 100 μg/ml heparin, with or without 1 μM NusG. Pausing reaction mixtures were incubated at 23°C, and aliquots were removed at various times. Transcription of the last aliquot was chased for 10 min at 37°C with a 0.5 mM concentration of each NTP. Transcription termination assays were performed as described for pausing except that the extension reaction mixtures were incubated at 37°C for 10 min. The 3′ ends of paused and terminated transcripts were mapped using sequencing reactions performed by *in vitro* transcription in the presence of one of four 3′ deoxynucleoside triphosphates (dNTPs). RNA bands were visualized with a phosphorimager and quantified using ImageQuant (GE Healthcare Life Sciences). Details of the procedures are described in Text S1.

### RNase T1 structure mapping.

*yxjB* RNA (+1 to +190) was synthesized with an RNAMaxx kit (Agilent Technologies). RNA was subjected to 5′ end labeling using T4 polynucleotide kinase (New England BioLabs) and [γ-^32^P]ATP. Labeled RNAs were renatured by heating for 1 min at 90°C followed by cooling to room temperature. Reaction mixtures (10 μl) contained 2 nM RNA, 10 mM Tris-HCl (pH 7.5), 10 mM MgCl_2_, 100 mM KCl, 40 ng of yeast RNA, 7.5% glycerol, 0.1 mg/ml xylene cyanol, and 200 μg/ml bovine serum albumin (BSA). RNA cleavage was performed by addition of 0.016 U RNase T1 (Thermo Fisher Scientific) followed by incubation for 15 min at 37°C. Reactions were stopped by adding 10 μl stop solution. Samples were heated for 5 min at 90°C and fractionated through standard 6% sequencing gels. Cleaved patterns were examined using a phosphorimager.

### β-Galactosidase assay.

B. subtilis cultures containing transcriptional or translational fusions were grown at 37°C in LB. When appropriate, growth media also contained 5 μg/ml chloramphenicol, 12.5 μg/ml kanamycin, 12.5 μg/ml tetracycline, and various concentrations of tylosin or erythromycin. β-Galactosidase activity was determined as described previously (33).

### Cell growth with tylosin.

The PLBS867 and PLBS868 strains were grown at 37°C in LB with 0 or 4 μg/ml tylosin with or without 0.5 mM IPTG (isopropyl-β-d-thiogalactopyranoside). A photograph of the culture tubes was taken after 20 h. *yxjB* overexpression was confirmed by primer extension of total cellular RNA extracted from the same strains. Strain PLBS338 was grown in LB media containing various tylosin concentrations until mid-exponential phase. Cultures were serially diluted 10-fold and then spotted onto LB plates containing various tylosin concentrations. Photographs of the plates were taken the next day.

### Toeprint of tylosin-induced ribosome stalling.

This analysis represents a modified version of a previously published procedure (11) performed using a PURExpress system (New England Biolabs). Briefly, a DNA template containing a T7 promoter and the *yxjB* leader region was added to PURExpress reactions with or without tylosin or erythromycin and incubated for 1 h at 37°C. A labeled DNA toeprint primer complementary to positions 140 to 166 of *yxjB* transcription was added to each reaction, and primer extension was carried out for 1 h at 37°C with SuperScript III. Reactions were terminated by the addition of stop solution. Samples were denatured prior to fractionation through 6% sequencing gels. Radioactive bands were visualized using a phosphorimager. Details of the procedure are described in Text S1.
