# Supplementary figures and images for: NusG-Dependent RNA Polymerase Pausing and Tylosin-Dependent Ribosome Stalling Are Required for Tylosin Resistance by Inducing 23S rRNA Methylation in Bacillus subtilis
Source: mBio. 2019 Nov 12;10(6):e02665-19. doi: 10.1128/mBio.02665-19 (PMC6851288; doi:10.1128/mBio.02665-19)

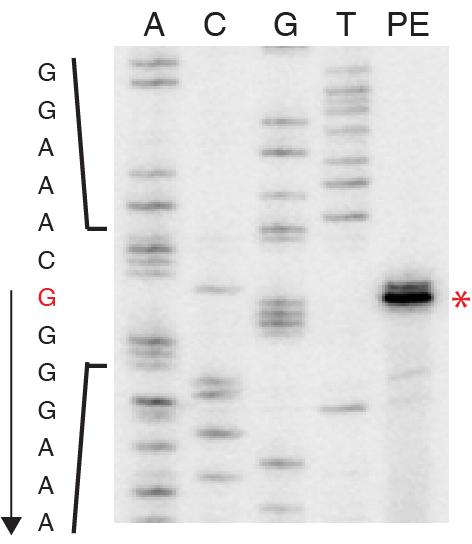

Supplement: FIG S1 [file mBio.02665-19-sf001.tif]

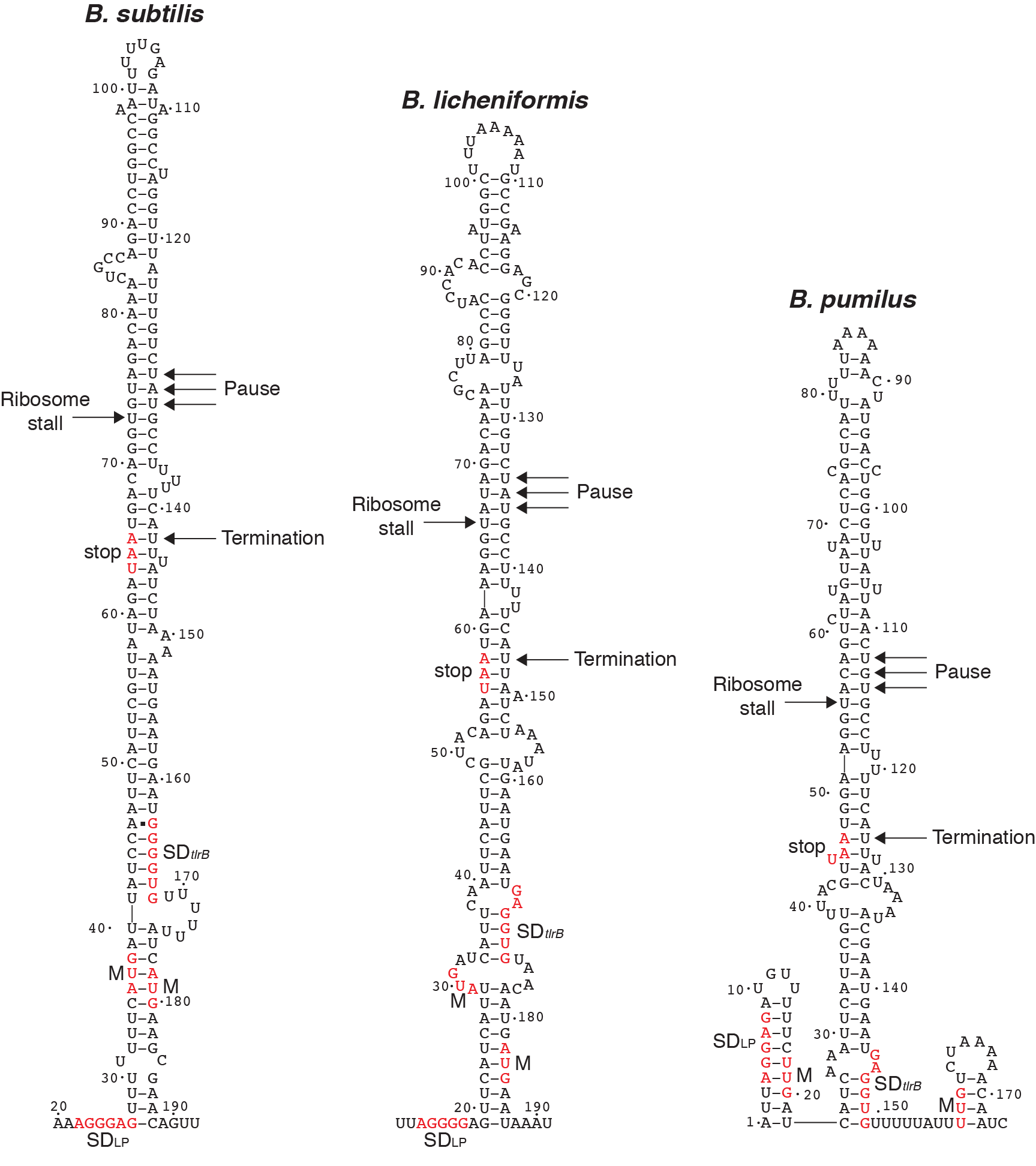

Supplement: FIG S2 [file mBio.02665-19-sf002.tif]

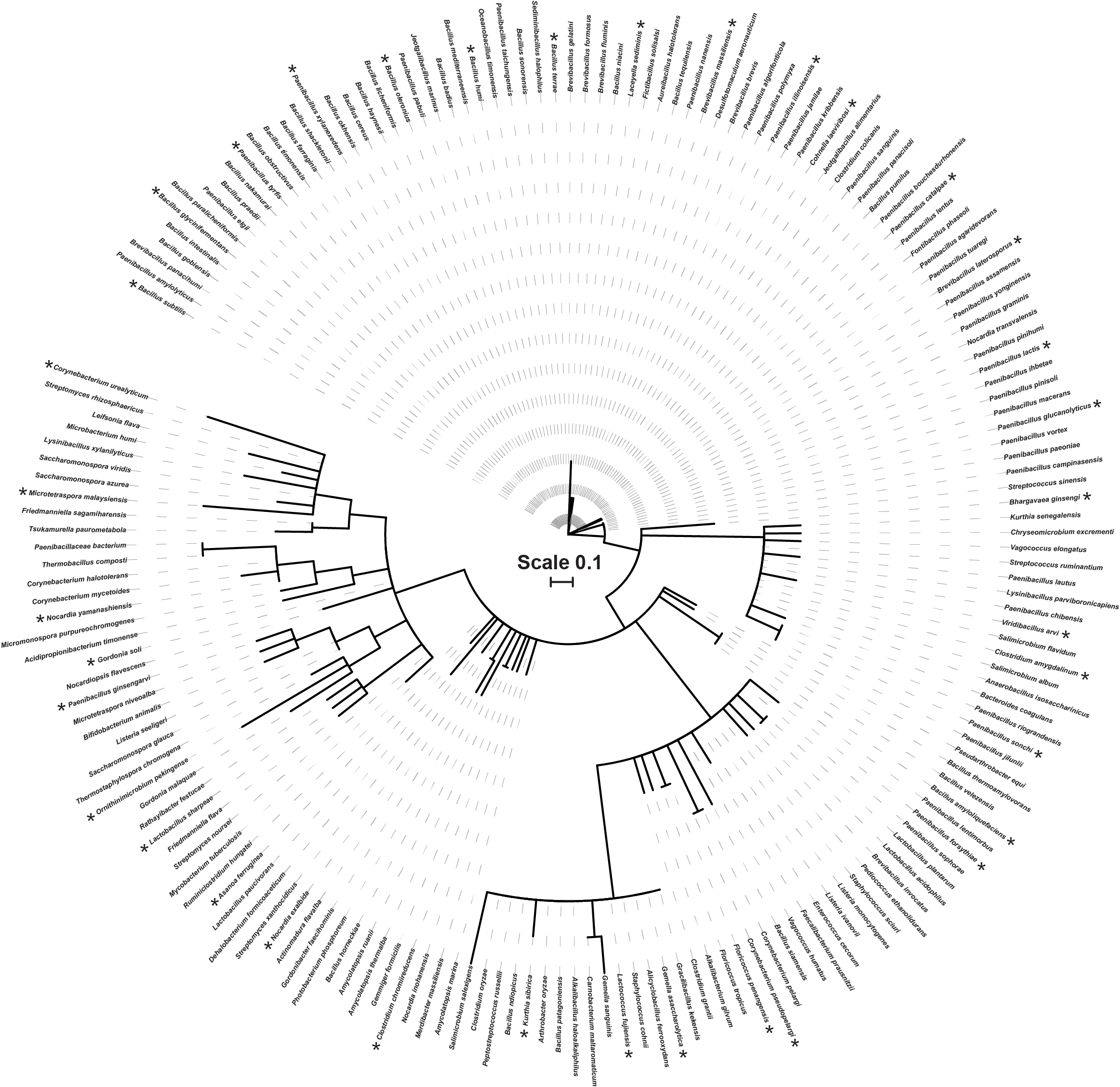

Supplement: FIG S3 [file mBio.02665-19-sf003.tif]

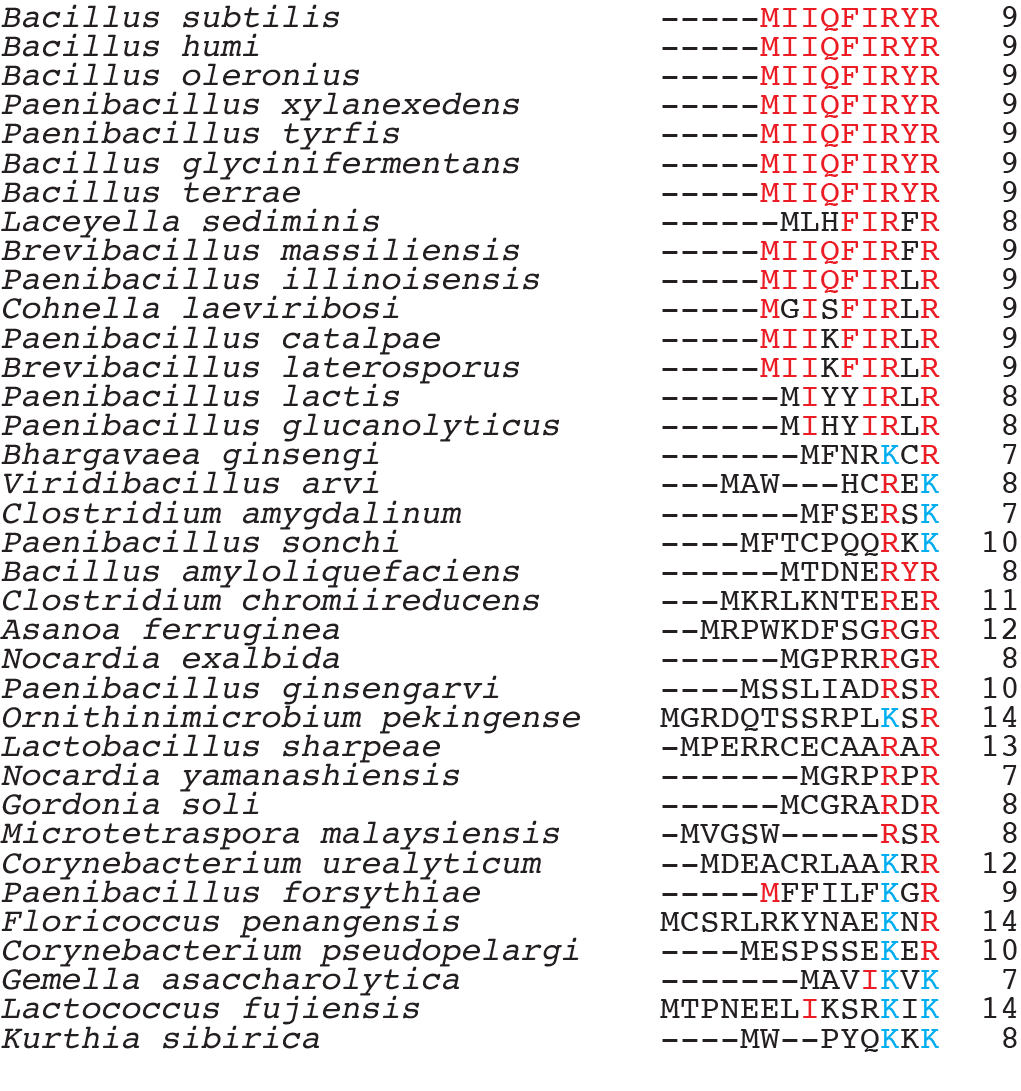

Supplement: FIG S4 [file mBio.02665-19-sf004.tif]
