# Supplementary material for: NusG-Dependent RNA Polymerase Pausing and Tylosin-Dependent Ribosome Stalling Are Required for Tylosin Resistance by Inducing 23S rRNA Methylation in Bacillus subtilis
Source: mBio. 2019 Nov 12;10(6):e02665-19. doi: 10.1128/mBio.02665-19 (PMC6851288; doi:10.1128/mBio.02665-19)
Supplement: TEXT S1 [file mBio.02665-19-s0001.docx]

## **SUPPLEMENTAL MATERIALS AND METHODS**

**Bacterial strains and plasmids.** Strain PLBS852 was constructed by transformation of PLBS338 with chromosomal DNA from BKE39010 (*yxjB::erm*) and selection for erythromycin resistance. Replacement of the WT *yxjB* allele was confirmed by PCR.

The cloning vector pTZ19R (Thermo Fisher Scientific) and the *B. subtilis* integration vectors ptrpBGI-PLK (1) and pDH32 (2) have been described. Plasmid pAY185 (pVector) is an *E. coli-B. subtilis* shuttle vector containing an IPTG-inducible P_T7A1_ promoter with an extended -10 sequence, *lacI*^q^, and Tc^r^. Plasmid pYH275 (pYxjB) was constructed by cloning a chromosomally-derived PCR fragment containing the *yxjB* coding sequence between the NdeI and BspEI sites of pAY185. PLBS338 was transformed with pYxjB and pVector to generate strains PLBS867 and PLBS868, respectively.

Plasmid pKM1 was constructed by cloning a PCR fragment containing -396 to +222 relative to the start of *yxjB* transcription into the EcoRI and HindIII sites of pTZ19R. Plasmid pYH263 containing the WT P*_yxjB_*-*yxjB'-'lacZ* translational fusion was constructed by subcloning the EcoRI-HindIII fragment from pKM1 into the same sites of ptrpBGI-PLK. After linearization of pYH263 with PstI, this fusion was integrated into the chromosomal *amyE* locus of strains PLBS338 and PLBS538 to generate strains PLBS800 and PLBS954, respectively. The LP start codon mutation (T37C) in the P*_yxjB_*-*yxjB'-'lacZ* translational fusion was generated in two steps. First, pYH327 was generated by PCR using mutagenic primers and plasmid pKM1 as the DNA template. Plasmid pYH328 was then constructed by subcloning the EcoRI-HindIII fragment from pYH327 into ptrpBGI-PLK. After linearization of pYH328 with PstI, this fusion was integrated into *amyE* locus of PLBS338 to produce PLBS957. The LP RYR to AYA motif mutation (C54G:G55C:T56C:A60G:G61C) in the P*_yxjB_*-*yxjB'-'lacZ* translational fusion was also generated in two steps. First, pYH335 was generated by PCR using mutagenic primers and plasmid pKM1 as the DNA template. pYH337 was then constructed by subcloning the EcoRI-HindIII fragment from pYH355 into ptrpBGI-PLK. After linearization of pYH337 with PstI, this fusion was integrated into the *amyE* locus of PLBS338 to create PLBS964. The pause site mutation (T131A) in plasmid pJJ3 was constructed by PCR using mutagenic primers and plasmid pKM1 as the DNA template. Plasmid pJJ6 was then generated by subcloning the EcoRI-HindIII fragment from pJJ3 into the same sites of ptrpBGI-PLK. After linearization of pJJ6 with PstI, this fusion was integrated into the *amyE* locus of PLBS338 to create PLBS959.

The WT P*_yxjB_*-*yxjB-lacZ* transcriptional fusion was constructed in two steps. First, a PCR fragment containing -396 to +153 relative to the start of *yxjB* transcription was cloned into the EcoRI and BamHI sites of pTZ19R, resulting in pBM12. In the second step, pBM13 was generated by subcloning the EcoRI-BamHI fragment from pBM12 into pDH32. After linearization of pBM13 with PstI, this fusion was integrated into *amyE* locus of PLBS338 to produce PLBS877. The terminator mutation (G71A:G72A) in the P*_yxjB_*-*yxjB-lacZ* transcriptional fusion was also generated in two steps. First, pJJ7 was generated by PCR using mutagenic primers and plasmid pBM12 as the DNA template. pJJ10 was then constructed by subcloning the EcoRI-BamHI fragment from pJJ7 into pDH32. After linearization of pJJ10 with PstI, this fusion was integrated into the *amyE* locus of PLBS338 to create PLBS960.

The P*_yxjB_*-LP'-'*lacZ* translational fusion in pJJ1 was constructed by cloning a PCR fragment containing -396 to +44 relative to the start of *yxjB* transcription into the EcoRI and HindIII sites of ptrpBGI-PLK. After linearization of pJJ1 with PstI, this fusion was integrated into the *amyE* locus of PLBS338 to produce PLBS952. Lastly, the template for single round *in vitro* transcription (pYH268) was constructed by subcloning +38 to +222 relative to the start of *yxjB* transcription into pAY66 (3) between the BamHI and HindIII sites.

**Primer extension assays.** For determination of the *yxjB* transcription start site, total cellular RNA was isolated from a late-exponential phase culture of *B. subtilis* PLBS338 grown in LB using the RNeasy kit (Qiagen). Ten μg of RNA was hybridized to 150 nM of a ^32^P-end-labeled oligonucleotide that was complementary to nt 54-74 of the *yxjB* leader. Reaction mixtures (10 μl) containing 2 µl of hybridization mixture, 375 µM dNTPs, 10 mM dithiothreitol, 200 µg/ml BSA, 1X SuperScript III buffer, and 1 µl SuperScript III Reverse Transcriptase (Thermo Fisher Scientific) were incubated for 15 min at 42°C. Reactions were terminated with 10 μl of stop solution (95% formamide, 20 mM EDTA, 0.025% SDS, 0.025% xylene cyanol, 0.025% bromophenol blue). Samples were denatured prior to fractionation through 6% polyacrylamide sequencing gels. Sequencing reactions were performed using pKM1 as a template and the same oligonucleotide as a primer. Radiolabeled bands were imaged on a Typhoon Variable Mode 8600 Phosphorimager (GE Healthcare Life Sciences).

For identification of 23S rRNA methylation sites, RNA was isolated from *B. subtilis* strains PLBS338, PLBS852, and PLBS867, and *E. coli strain* MG1655 grown in LB. Two μg of total RNA was hybridized to 2 pmol of a ^32^P-end-labeled oligonucleotide complementary to nt 798-818 of *E. coli* 23S rRNA; this region differs by only one nucleotide in *B. subtilis* 23S rRNA (nt 845-865). RNA extraction, RT reactions, gel running, and analysis were identical to those described above except that primer extension was for 30 min.

**Two-step single-round *in vitro* transcription assays.** DNA templates were PCR-amplified from plasmids containing WT or mutant *yxjB* leader sequences driven by a consensus σ^A^ promoter with an extended -10 sequence, and a 29-nt C-less cassette (pYH268 and its derivatives). In the first step, halted elongation complexes containing a 29-nt transcript were formed for 5 min at 37°C in a 20 µl reaction containing 50 to 100 nM DNA template, ATP and GTP (40 µM each), 1 µM UTP, 50 µg/ml acetylated BSA, 75 µg/ml (0.19 µM) *B. subtilis* RNAP holoenzyme, 0.38 µM SigA, and 1 µCi of [α-^32^P]UTP at 37°C (no CTP). RNAP and σ^A^ were added from a 20x stock solution containing 1.5 mg/ml RNAP and 0.35 mg/ml σ^A^ in enzyme dilution buffer (20mM Tris-HCl [pH 8.0], 40 mM KCl, 1 mM DTT, and 50% glycerol). Halted complexes were diluted 2- to 4-fold with 1x transcription buffer containing 100 µg/ml acetylated BSA, and KCl such that the final KCl concentration was 17 mM. Elongation was resumed by the addition of all four NTPs, 100 µg/ml heparin, ± 1 µM NusG. The final NTP concentrations were 150 µM. Pausing reactions were incubated at 23°C in reaction mixtures containing 10 mM KCl (3 volumes of diluted halted elongation complexes plus 2 volumes of the NTP, heparin, and NusG-containing extension mix). Aliquots were removed at various times. Transcription of the last aliquot was continued for 10 min at 37°C with 0.5 mM each NTP (chase reaction). Transcription termination assays were performed as described for pausing except that extension reactions were incubated at 37°C for 10 min in the presence of 20 mM KCl, and 1 µM NusA was used instead of NusG. 3’ ends of paused and terminated transcripts were mapped using sequencing reactions performed by single-round *in vitro* transcription as described above in the presence of one out of four 3’-deoxynucleotide triphosphates. Transcription was stopped by addition of an equal volume of the gel loading solution (40 mM Tris-base, 20 mM EDTA [pH 8.0], 0.2% SDS, 0.05% bromophenol blue, and 0.05% xylene cyanol in formamide). Samples were fractionated through standard 5% sequencing gels. RNA bands were visualized with a phosphorimager and quantified using ImageQuant software (GE Healthcare Life Sciences). Experiments were performed at least twice with comparable results.

**Toeprint of tylosin-induced ribosome stalling.** This analysis is a modified version of a previously published procedure (4). PCR fragments containing a T7 RNAP promoter and -24 to +180 relative to *yxjB* transcription was produced using pKM1 and pYH335 as DNA templates for the WT and AYA mutant leaders, respectively. pKM1 was also used for generating a DNA sequencing ladder using the Sequenase v. 2.0 DNA sequencing kit (Thermo Fisher Scientific). PCR fragment purification was done with the Qiaquick PCR purification kit (QIAGEN) with water elution. Each reaction (5 µl) contained 10 ng PCR fragment, 2 µl solution A (PURExpress, New England Biolabs**)**, 1.5 µl solution B, water or tylosin (5 μM). Reaction mixtures were incubated for 1 hr at 37°C. A DNA toeprint primer complementary to +140 to +166 relative to *yxjB* transcription was 5’-end-labeled with γ-^32^P[ATP] and T4 polynucleotide kinase, purified using a mini quick spin oligo column (Roche), and then eluted with 30 µl TE. Two-µl of the 5’-end-labeled primer was added to each toeprint reaction, and then incubated for 3 min at 55°C. Following the addition of 8 µl primer extension mixture, the resulting mixture was incubated for 1 hr at 37°C. The primer extension mixture for one reaction contained: 3 µl 5X FS buffer for Superscript III, 1.5 µl 0.1 mM dithiothreitol, 1.5 µl 2.5 mM dNTP, 0.375 µl RNasin (Promega), 1.125 µl water, and 0.5 µl Superscript III. Reactions were terminated by the addition of 15 μl stop solution. Samples were heated for 2 min at 95°C prior to fractionation through standard 6% polyacrylamide sequencing gels. Radioactive bands were visualized using a phosphorimager.

**Multiple sequence alignment and phylogeny.** The maximum likelihood phylogeny was constructed from the result of a multiple sequence alignment analysis conducted on 180 Gram-positive organisms containing leader peptides predicted *in silico* to both be located directly upstream of *yxjB* homologs, and to contain the R/K-X-R/K ribosome stalling motif at the C-terminus (4). The top 15,000 *yxjB* homologs were identified using BLASTp, the multiple sequence alignment analysis was conducted via MUSCLE, and the phylogeny was computed on the MEGA7 platform, all of which operated under standard statistical parameters (5-7, https://blast.ncbi.nlm.nih.gov/). The custom *in silico* leader peptide prediction software was written in python. This software takes the output of the BLASTp analysis and locates the *yxjB* homolog in the reference genome of each respective organism iteratively using tBLASTn. After this, the 300 nt directly upstream of each homolog was translated in all three reading frames. For each respective frame, the regular expression ‘[K|R].[K|R]$’ was used to search for the putative ribosomal stalling motif. To identify the likely N-termini of each leader peptide, the closest upstream methionine was identified via the known microbial N-terminal methionine encoding preferences (AUG >> GUG > UUG > CUG) (8). In circumstances where multiple viable leader peptides were identified, the leader peptide closest to the start codon of the *yxjB* homolog was selected. Tree annotation and display was created with the interactive tree of life web platform (iTOL), and the multiple sequence alignment plot was created with the R statistical graphing software (9). Custom scripts used for this work are available upon request.

**SUPPLEMENTAL REFERENCES**

1. Merino E, Babitzke P, Yanofsky C. 1995. *trp* RNA-binding attenuation protein (TRAP)- *trp* leader RNA interactions mediate translational as well as transcriptional regulation of the *Bacillus subtilis trp* operon. J Bacteriol 177:6362–6370.

2. Grandoni JA, Fulmer SB, Brizzio V, Zahler SA, Calvo JM. 1993. Regions of the *Bacillus subtilis ilv-leu* operon involved in regulation by leucine. J Bacteriol 175:7581– 7593.

3. Yakhnin AV, Yakhnin H, Babitzke P. 2008. Function of the *Bacillus subtilis* transcription elongation factor NusG in hairpin-dependent RNA polymerase pausing in the *trp* leader. Proc Natl Acad Sci U S A. 105:16131–16136.

4. Davis AR, Gohara DW, Yap MN. 2014. Sequence selectivity of macrolide-induced translational attenuation. Proc Natl Acad Sci U S A 111:15379–15384.

5. Altschul SF, Gish W, Miller W, Myers EW, Lipman DJ. 1990. Basic local alignment search tool. J Mol Biol 215:403–410.

6. Kumar S, Stecher G, Tamura K. 2016. MEGA7: molecular evolutionary genetics analysis version 7.0 for bigger datasets. Mol Biol Evol 33:1870–1874.

7. Chojnacki S, Cowley A, Lee J, Foix A, Lopez R. 2017. Programmatic access to bioinformatics tools from EMBL-EBI update: 2017. Nucleic Acids Res 45:W550-W553.

8. Belinky F, Rogozin IB, Koonin EV. 2017. Selection on start codons in prokaryotes and potential compensatory nucleotide substitutions. Sci Rep 8:9260.

9. Letunic I, Bork P. 2016. Interactive tree of life (iTOL) v3: an online tool for the display and annotation of phylogenetic and other trees. Nucleic Acids Res **8:**W242-W245.
