# Supplementary material for: NusG-Dependent RNA Polymerase Pausing and Tylosin-Dependent Ribosome Stalling Are Required for Tylosin Resistance by Inducing 23S rRNA Methylation in Bacillus subtilis
Source: mBio. 2019 Nov 12;10(6):e02665-19. doi: 10.1128/mBio.02665-19 (PMC6851288; doi:10.1128/mBio.02665-19)
Supplement: TABLE S1 [file mBio.02665-19-st001.docx]

**Table S1.** Plasmids used in this study.

| Plasmid | Description^a^ | Source |
| --- | --- | --- |
| pTZ19R | cloning vector | Thermo Fisher Scientific |
| ptrpBGI-PLK | translational fusion integration vector | 1 |
| pDH32 | transcriptional fusion integration vector | 2 |
| pAY66 | cloning vector for *in vitro* transcription | 3 |
| pAY185 (pVector) | *E. coli-B. subtilis* shuttle vector with IPTG-inducible P_T7A1_ and *lacI*^Q^ | this study |
| pYH275 (pYxjB) | *yxjB* coding sequence in pAY185 | this study |
| pKM1 | *yxjB* promoter and leader (-396 to +222) in pTZ19R | this study |
| pYH263 | P*_yxjB_*-*yxjB'-'lacZ* translational fusion (-396 to +222) in ptrpBGI-PLK | this study |
| pYH327 | *yxjB* promoter and leader (-396 to +222) in pTZ19R [T37C] | this study |
| pYH328 | P*_yxjB_*-*yxjB'-'lacZ* translational fusion (-396 to +222) in ptrpBGI-PLK [T37C] | this study |
| pYH335 | *yxjB* promoter and leader (-396 to +222) in pTR19R [C54G:G55C:T56C:A61G:G62C] | this study |
| pYH337 | P*_yxjB_*-*yxjB'-'lacZ* translational fusion (-396 to +222) in ptrpBGI-PLK [C54G:G55C:T56C:A61G:G62C] | this study |
| pJJ3 | *yxjB* promoter and leader (-396 to +222) in pTZ19R [T131A] | this study |
| pJJ6 | P*_yxjB_*-*yxjB'-'lacZ* translational fusion (-396 to +222) in ptrpBGI-PLK [T131A] | this study |
| pBM12 | *yxjB* promoter and leader (-396 to +153) in pTZ19R | this study |
| pBM13 | P*_yxjB_*-*yxjB-lacZ* transcriptional fusion (-396 to +222) in pDH32 | this study |
| pJJ7 | *yxjB* promoter and leader (-396 to +153), G71A:G72A in pTZ19R | this study |
| pJJ10 | P*_yxjB_*-*yxjB-lacZ* transcriptional fusion, G71A:G72A in pDH32 | this study |
| pJJ1 | P*_yxjB_*-LP*'-'lacZ* translational fusion (-396 to +44) in ptrpBGI-PLK | this study |
| pYH268 | vector for *in vitro* transcription (+38 to +222) in pAY66 | this study |

^a^cloned *yxjB* sequences are indicated in ( ). *yxjB* leader mutations are indicated in [ ].
