# Supplementary material for: NusG-Dependent RNA Polymerase Pausing and Tylosin-Dependent Ribosome Stalling Are Required for Tylosin Resistance by Inducing 23S rRNA Methylation in Bacillus subtilis
Source: mBio. 2019 Nov 12;10(6):e02665-19. doi: 10.1128/mBio.02665-19 (PMC6851288; doi:10.1128/mBio.02665-19)
Supplement: TABLE S2 [file mBio.02665-19-st002.docx]

**Table S2.** Oligonucleotides used in this study.

| Oligonucleotide | Sequence | Use |
| --- | --- | --- |
| yxjB EcoRI For | GGCGAATTCTTATCCTTCACAAATTCTGGA AGTG | *yxjB* translational fusion  *yxjB* transcriptional fusion  LP translational fusion |
| TLN yxjB HindIII Rev | GGCAAGCTTGCAGAGCGGGCATCGAAACAT GC | *yxjB* translational fusion |
| TXN yxjB BamHI Rev | CTGGGGATCCTTTTTAGATAAATGAAAAAG  GCATAGACAAATAAACC | *yxjB* transcriptional fusion |
| LP yxjB HindIII Rev | GGGCAAGCTTGATAATCATGAAAAAAACTC CCTTTCTGC | LP translational fusion |
| GG to AA For | GTTATAGATAATGACAAATGTAGACAAACT  GCC | Terminator mutation, G71A:G72A |
| GG to AA Rev | GGCAGTTTGTCTACATTTGTCATTATCTAT  AAC | Terminator mutation, G71A:G72A |
| T131A For | AGCCTTTTTCATTTATCTAAAAATG | Pause mutation, T131A |
| T131A Rev | TAGACAAATAAACCTAGGCCTATC | Pause mutation, T131A |
| LP start For | GAAAGGGAGTTTTTTTCACGATTATCCAAT TCATTCG | LP start codon mutation,  T37C |
| LP start Rev | CGAATGAATTGGATAATCGTGAAAAAAACT CCCTTTC | LP start codon mutation,  T37C |
| RYR mut For | GATTATCCAATTCATTGCCTATGCATAATG ACAGGTGTAG | LP RYR to AYA mutation,  C54G:G55C:T56C:A61G:G62C |
| RYR mut Rev | CTACACCTGTCATTATGCATAGGCAATGAA TTGGATAATC | LP RYR to AYA mutation,  C54G:G55C:T56C:A61G:G62C |
| T7yxjB PstI For | AGCCTGCAGTAATACGACTCACTATAGGGA TATTTGTTGTATGATGGAAACGGGG | Ribosome toeprint,  template for RNA |
| yxjB HindIII Rev | GGCAAGCTTGCAGAGCGGGCATCGAAACAT GC | Ribosome toeprint,  template for RNA |
| Toeprint 3 | CCCCATTCATTCATTTTTAGATAAATG | Ribosome toeprint,  primer extension |
| PE2 | CACCTGTCATTATCTATAACG | *yxjB* transcription start,  primer extension |
| yxjB NdeI For | CCGCATATGAAGCGAACAGTTGATTTCAGC ATGTTTCGA | pYxjB |
| yxjB BspEI Rev | GCCCTCGGATCATTTCATTCCTATTAGGAT ATCAACATCAACCG | pYxjB |
| BamHI For | GGATCCGATTATCCAATTCATTCGTTATAG ATAATGACAGG | Template for single round transcription |
| HindIII Rev | GCCAAGCTTGCAGAGCGGGCATCGAAAC | Template for single round transcription |
| PSL | CAGCTTGACAAATACACAAGAGTGTGTTAT AATGCAATTAG | Template for single round transcription |
| T7 TXN Start For | GAAATAATACGACTCACTATAGGGGAAACC  CATACAGCAG | Template for generating RNA for structure mapping |
| yxjB Structure Map Rev | CTGTTCGCTTCATGATAAAAAACACCCC | Template for generating RNA for structure mapping |
| yxjB Rev | GCAGAGCGGGCATCGAAACATGCTG | Template for single round transcription |
| 23S yxjB | CGGGGAGAACCAGCTATCTCC | Primer extension of 23S rRNA |
